# Application of the New Classification on Patients with a Disorder of Sex Development in Indonesia

**DOI:** 10.1155/2012/237084

**Published:** 2011-12-29

**Authors:** A. Zulfa Juniarto, Yvonne G. van der Zwan, Ardy Santosa, Remko Hersmus, Frank H. de Jong, Renske Olmer, Hennie T. Bruggenwirth, Axel P. N. Themmen, Katja P. Wolffenbuttel, Leendert H. J. Looijenga, Sultana M. H. Faradz, Stenvert L. S. Drop

**Affiliations:** ^1^Department of Human Genetics, Center for Biomedical Research, Faculty of Medicine Diponegoro University (FMDU), Semarang 50321, Indonesia; ^2^Division of Pediatric Endocrinology, Department of Pediatrics, Sophia Children's Hospital, Erasmus MC, P.O. Box 2060, 3000 CD Rotterdam, The Netherlands; ^3^Department of Urology, Dr. Kariadi Hospital, Semarang 50321, Indonesia; ^4^Department of Pathology, Josephine Nefkens Institute, Erasmus University Medical Center, 3015 CE Rotterdam, The Netherlands; ^5^Section of Endocrinology, Department of Internal Medicine, Erasmus University Medical Center, 3015 CE Rotterdam, The Netherlands; ^6^Department of Clinical Genetics, Erasmus University Medical Centre, 3015 CE Rotterdam, The Netherlands; ^7^Department of Paediatric Urology, Erasmus University Medical Centre, 3015 CE Rotterdam, The Netherlands

## Abstract

Disorder of sex development (DSD) patients in Indonesia most often do not receive a proper diagnostic evaluation and treatment. This study intended to categorize 88 Indonesian patients in accordance with the new consensus DSD algorithm. Diagnostic evaluation including clinical, hormonal, genetic, imaging, surgical, and histological parameters was performed. Fifty-three patients were raised as males, and 34 as females. Of 22 patients with 46, XX DSD, 15 had congenital adrenal hyperplasia, while in one patient, an ovarian Leydig cell tumor was found. In all 58 46, XY DSD patients, 29 were suspected of a disorder of androgen action (12 with an androgen receptor mutation), and in 9, gonadal dysgenesis was found and, in 20, severe hypospadias e.c.i. Implementation of the current consensus statement in a resource-poor environment is very difficult. The aim of the diagnostic workup in developing countries should be to end up with an evidence-based diagnosis. This is essential to improve treatment and thereby to improve the patients' quality of life.

## 1. Introduction

The sequential expression of many genes is essential for gonadal development in the male as well as in the female [1, 2]. In addition, timely secretion and action of hormones such as androgens and anti-Müllerian hormone (AMH) are crucial for normal male development [3]. Mutation analysis of genes related to these factors in patients with genital disorders has substantiated their essential role [4–7]. Therefore, in a number of cases, a specific diagnosis can be made by mutation analysis. Disorders of sex development (DSD) are defined as congenital conditions in which development of chromosomal, gonadal, or anatomical sex is atypical [8]. In patients categorized under the term 46, XY or 46, XX DSD with anomalies of gonadal development, often no specific etiology can be established [9]. Yet the establishment of a specific diagnosis is relevant with regard to proper gender assignment as well as regarding hormonal and surgical treatment. Moreover, patients with various forms of 46, XY DSD and chromosomal DSD are at a substantially increased risk of developing gonadal germ cell tumors [10]. 

In Indonesia, patients presenting with DSD most often do not receive a diagnostic evaluation and there are limited options for medical and surgical treatment. In this study, we performed a diagnostic evaluation in 88 patients with DSD referred to a major Centre in Semarang, Indonesia. The aim of this study was to categorize the patients in accordance with the nomenclature proposed in the new consensus statement [8]. Therefore, we performed a diagnostic evaluation including clinical and hormonal parameters. Furthermore, results from imaging and surgery as well as genetic and histological parameters were evaluated. 

## 2. Subject and Methods

### 2.1. Subjects

Eighty-eight patients (from 84 index patients) with various forms and degrees of DSD were evaluated consecutively. They were referred for chromosomal analysis by clinicians of the departments of Urology, Pediatrics, Internal Medicine, and Obstetrics of the Dr. Kariadi Hospital, Semarang, Indonesia. Referral took place between 2004 and 2006 to the department of Human Genetics Center for Biomedical Research, Faculty of Medicine Diponegoro University (FMDU), Semarang. Reason of referral was the presence of ambiguous genitalia or any anatomical abnormality of external or internal genitalia, including penoscrotal hypospadias, with or without descended testes. Patients with sex chromosome aberrations were included except patients with classical Klinefelter (47, XXY) and Turner syndromes (45, XO). In addition, four patients with cloacal malformation were excluded.

All patients were recalled to the hospital for a physical examination, pedigree construction, and collection of blood for hormonal and gene mutation analyses. The age at initial presentation was also the age of investigation and the start of followup in 87 patients, and one patient with presumptive CAH already received suppletion therapy for 9 months. The local medical ethics committee approved this study, and informed consent was obtained from all participants, their parents, or guardians. 

### 2.2. Methods

**Figure 1:**
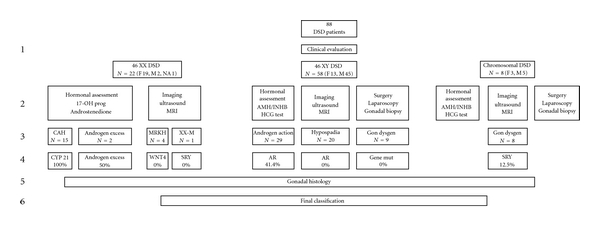
Stepwise diagnostic approach. An algorithm used with the aim of classifying the patients following the new classification system [8]. Row number 1 represents the clinical evaluation of the patients and classification following the primary root; number 2 the hormonal analysis and imaging followed by the secondary root classification (row 3). Row number 4 shows the percentage mutations that were found and number 5 the gonadal histology leading to the tertiary root and final classification of the patients. Explanation of abbreviations: MRKH: Mayer-Rokitansky-Küstner-Hauser Syndrome; XX-M; XX male.

A stepwise diagnostic approach (Figure 1) was used in order to determine the diagnosis in each patient. First of all the patients were clinically evaluated, a detailed description of the external genitalia was obtained, and the genitalia were staged according to Quigley at al. [11]. The assigned gender was also recorded. Subsequently, chromosomal patterns were determined, and, based on the results, the patients were categorized according to the primary root of the recent classification [8]: 46, XY DSD, 46, XX DSD, or chromosomal DSD. In all patients, a blood sample was obtained for hormonal and gene analysis; in patients with 46, XY DSD, or Y containing chromosomal DSD, an additional blood sample was obtained 72 hrs after the intramuscular injection of 1500 IU hCG (Pregnyl Organon, Oss, The Netherlands). The hCG test was not performed in 11 patients for the following reasons: the gonads had been removed (*n* = 3), logistic reasons (*n* = 7) and, in one patient, hCG therapy had been started by the referring doctor in order to enlarge the penis. Subsequently, imaging was offered to all the patients as well as surgery in the form of a laparoscopy or cystoscopy whenever needed for diagnostic options or for gonadectomy in case of a high tumor risk. Based on the results, a differential diagnosis was made followed by gene mutation analysis. Finally, gonadal samples were analyzed when they were available in order to complete the classification.

#### 2.2.1. Chromosome Analysis

Karyotype was established using a G-banding technique in the Molecular and Cytogenetics Laboratory of the Center for Biomedical Research of FMDU (Semarang). G banding was also performed for confirmation of the presence of the Y chromosome.

#### 2.2.2. Serum Hormones

Serum determinations of inhibin B, AMH, LH, and FSH in the basal serum samples were performed in the endocrine laboratory of Erasmus MC (Rotterdam) as described previously [12]. Testosterone was determined in serum collected before and after injection of hCG using the Coat-a-Count radioimmunoassay purchased from Siemens (Los Angeles, Calif) [12]. Androstenedione, dehydroepiandrosterone sulphate, and progesterone were also measured in these samples using the Immulite 2000 (Siemens). Finally, 17-hydroxyprogesterone levels were estimated using an in-house method [13]. Reference values were used as described earlier [12, 14].

#### 2.2.3. Gene Analysis

DNA was extracted from leucocytes of EDTA blood using the salting-out method as described earlier [15]. Based on the clinical and hormonal information, specific genes were analysed such as *CYP21A2* [16], and *LHR* [6]. *AR, SRY,* and *WNT4* were analysed by direct sequence analysis of the coding exons and exon-flanking intronic regions (reference sequence *AR *[17, 18]*:* nm_000044.2 numbering according to Gottlieb et al. [18], *SRY*: X53772.1 and *WNT4*: nm 030761.4).

#### 2.2.4. Pathology

Histopathological assessments were performed by means of hematoxylin and eosin stainings and immunohistochemistry for various markers of germ cells, for example, OCT3/4, TSPY, VASA, SCF (including double staining for OCT3/4-TSPY or VASA); as well as SOX 9 and FOXL2 for supportive cells [19]. 

## 3. Results

For a stepwise diagnostic approach, the algorithm shown in Figure 1 was followed. 

### 3.1. Clinical Evaluation and Chromosome Determination

All 88 patients were categorized according to their karyotype; there were 22 patients with 46, XX DSD, 58 patients with 46, XY DSD, and eight patients with chromosomal DSD.

**Figure 2:**
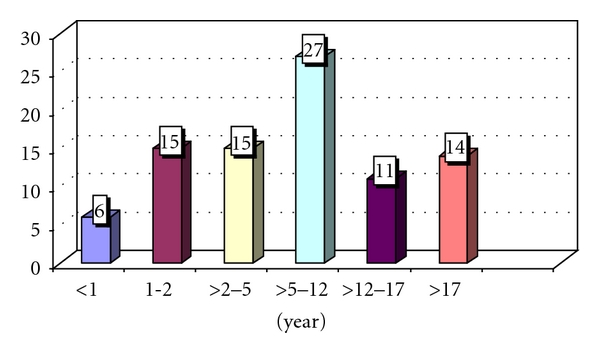
Age distribution of the patients at presentation.

Data on age, sex of rearing, and Quigley stage [11] of the 88 patients are provided in Table 1. The majority of patients were older than two years at the time of referral; only six patients were referred below the age of one year (Figure 2). The group of 88 patients contains four families with two or more patients with DSD (a total of nine). There was no known consanguinity of the parents. Fifty-two patients were raised as male and 35 as female,, while one patient's gender was undetermined due to early age; 16 patients (19%) were raised discordant with their genotype (two males with karyotype 46, XX and 14 females with karyotype 46, XY). Two 46, XY patients changed gender from female into male during the study, and one patient with a 46, XX karyotype decided to live as a male. One patient with presumed androgen excess died one week after inclusion without gender assignment. 

### 3.2. Hormonal Analysis, Imaging, and Surgery

#### 3.2.1. 46, XX DSD

Hormonal analysis showed that out of the 22 46, XX patients, 15 patients (68%) had serum values of adrenal steroids suggesting CAH. Fourteen of these 15 patients showed marked elevation of levels of 17-hydroxyprogesterone, androstenedione, and testosterone. In the remaining patient, these values were in the normal range as she already received corticosteroid suppletion therapy before referral. No patients with the salt wasting form of CAH were found. As expected, AMH and inhibin B levels were all in normal range for females (data not shown). In one patient with no history of steroid medication, adrenal steroid levels including cortisol were suggestive of cortisol resistance. This could not be confirmed by sequencing the glucocorticoid receptor gene, however.

Extremely high testosterone levels (basal level of 59.9 nmol/L) and slightly increased values of gonadotropins (LH: 12.3 IU/L, FSH: 8.1 IU/L) were identified in one 33-year-old patient with Quigley stage 4, phallus length 2.5 cm, and a low voice. Four patients had normal ovarian and adrenal hormone levels and, in view of the clinical presentation, were suspected of having the Mayer-Rokitansky syndrome [20].

One 11-year-old patient with the 46, XX male syndrome with palpable gonads in the scrotum had normal FSH and testosterone levels but low LH, AMH, and inhibin B levels for boys of this age. Testosterone rose from 0.3 to 3.4 nmol/L in response to hCG, suggesting that there were functional Leydig cells. 

Only in 12 of the 22 patients that were eligible, a diagnostic ultrasound was performed. In seven patients, surgery was advised, and again only in two (28%) patients, diagnostic surgery in the form of a laparoscopy or cystoscopy was done. The remaining patients refused because of economic reasons.

#### 3.2.2. 46, XY DSD

In all 58 patients, basal hormonal measurements were obtained. LH levels were elevated in nine patients, decreased in 35 patients, and normal for age in 14. FSH levels were elevated in 18 patients and in the normal range in the remaining 40. Finally, testosterone levels were elevated in 15 patients, decreased in 36 patients, and normal in seven patients.

Twelve patients showed elevated levels of AMH, 27 patients had levels in the normal range, and 17 patients had decreased levels of AMH. In one patient, after gonadectomy, AMH was not determined. 

Inhibin B levels were elevated in 17 patients, normal in 27 patients, and decreased in 14 patients. An hCG test was performed in 47/58 patients (see methods), and 45 patients showed a sufficient response of testosterone and its precursors. In two patients with a decreased response, Leydig cell hypoplasia was suspected. Due to lack of material, we could not test for DHT.

Diagnostic ultrasound was performed in 11 out of 58 (19%) patients, and, only in five (9%) patients, diagnostic procedures in the form of a cystoscopy were performed. In two (9%) of the 21 eligible patients, a gonadectomy was performed. The remaining patients refused because of economic and cultural reasons.

#### 3.2.3. Chromosomal DSD

The karyotype of the eight patients is provided in Table 2. The basal level of LH was increased for age in one patient. The level of FSH was elevated in two patients (age 2 and 33 years) with low levels of inhibin B in comparison with male values, while AMH and inhibin B were increased in comparison to normal female levels. 

Ultrasound was performed in five out of eight (62.5%) patients. In one (12.5%) patient a diagnostic procedure in the form of a cystoscopy was done. In only one (12.5%) of the eight eligible patients a gonadectomy was performed. The remaining patients refused because of economic and cultural reasons. Efforts to followup on these patients have been performed.

### 3.3. Secondary Root Classification

Based on the hormonal evaluation, imaging, and diagnostic surgery, the following secondary root categorization was made.

#### 3.3.1. 46, XX DSD

15 patients were suspected of congenital adrenal hyperplasia, one patient of a glucocorticoid receptor defect, four patients with Mayer-Rokitansky Syndrome, one patient with XX male syndrome, and one patient with ovotesticular DSD. 

#### 3.3.2. 46, XY DSD

29 patients were suspected of a disorder of androgen action, nine with a disorder of gonadal development, and 20 with severe hypospadias. We did not establish the diagnosis testosterone synthesis disorders in any of the patients, but we cannot rule out the presence of 5-alpha-reductase deficiency in our patients without a definitive diagnosis and a normal response of 5*α*-dihydrotestosterone (DHT) levels to hCG.

#### 3.3.3. Chromosomal DSD

All patients were categorized under the second root diagnosis disorders of gonadal development (gonadal dysgenesis). 

### 3.4. Mutation Analysis

Based on these results, mutation analysis was performed. 

#### 3.4.1. 46, XX DSD

Based on their phenotype, hormonal, and chromosomal analysis, *CYP21A2* analysis was performed in 15 patients, and indeed *CYP 21* gene mutations were found in all of them [16]. In four patients, clinically suspected of having Mayer-Rokitansky syndrome, *WNT4* gene analysis was negative. One patient was suspected of a glucocorticoid receptor defect; however, a mutation in the sequence of this receptor was not found.

#### 3.4.2. 46, XY DSD

Mutation analysis of the androgen receptor (AR) gene was performed in 29 patients who were suspected of having a disorder of androgen action and in 20 patients with severe hypospadias with a normal response to hCG. In two index patients (four patients), pathogenic AR mutations were found, R840H and 902insA. In an additional two patients, the sequence variant V730M was found, of which it is unlikely that it is causing the phenotype; functional studies showed that this variant is an activating mutation. It has been described as a somatic variant in patients with prostate cancer [21, 22]. In five index patients (six patients), unclassified variants were found, I603N, 2170T>A; P671S, 2373C>T; C175G, 885T>G; Q738R, 2575A>G; only one of these four variants (C175G) has been described at the nucleotide level. The three novel sequence variants (I603N, P671S, and Q738R) were functionally investigated [23]. Further mutation analysis for the SRD5A2 should be performed for patients without AR gene mutation.

In the 20 patients with severe hypospadias, no AR mutations were found. 

Two patients suspected of having Leydig cell hypoplasia were analyzed for a mutation in the LH receptor gene, but no mutation was found.

#### 3.4.3. Chromosomal DSD

An SRY deletion was found in one patient with mosaic Klinefelter XX/XXY. In the remaining seven patients, no SRY deletions were detected. 

### 3.5. Gonadal Histology

Histology of the gonads was available in four patients as shown in Table 3: one with 46, XX DSD (biopsy as mentioned earlier), two with 46, XY and one with chromosomal DSD (46, XY/46, XX). 

One patient with 46, XX DSD had an ovarian Leydig cell tumor. In this patient, ultrasonography did not reveal abnormalities. However, a diagnostic laparoscopy showed normal adrenal glands and large ovaries. During laparoscopy, a biopsy was obtained. In a 23-year-old 46, XY patient, the testis showed Leydig cell hyperplasia and atrophy of most seminiferous tubules but no evidence of CIS. A thirteen-year-old 46, XY boy was found to have Carcinoma *in situ* (CIS), the precursor lesion for malignant germ cell tumors, as reported recently [24]. Ovarian tissue with multiple cysts including primordial follicles and granulosa cells was found without evidence of malignancy in one patient with mosaic XX/XY. 

### 3.6. Final Classification

Based on the above-mentioned steps in the diagnostic workup in patients with DSD syndromes, a final classification was made following the current consensus statement [8]. Data are shown in Table 4. In sixteen 46, XX DSD patients (72%), a tertiary root classification was made, in patients with 46, XY DSD, this was the case in 12 patients (21%), and, in the group of patients with chromosomal DSD, a tertiary root classification was made in one patient (12.5%). Of course, the last category is a special one because the chromosomal abnormalities itself are an explanation for the etiology. In the remaining patients without an identified genetic or pathologic cause of DSD, the tertiary root category had to be “other.”

The 6 patients aged less than one year were diagnosed as follows: androgen action disorder (2), excess androgen (2), and unknown male undermasculinization (2). 

The two 46, XY patients who changed gender from female to male had the final diagnosis of androgen action disorder, while one 46,XX CYP21A2-deficient patient decided to live as a male. 

## 4. Discussion

Reports on presentation and age distribution of DSD patients in Asian countries are scarce and are mostly limited to CAH patients [25–27]. The age of presentation of the DSD patients in our study differs greatly from the age of presentation in the western world. More than 75% of the patients were over two years old. In India, 58% of the patients are referred within the first year of life [26]. Reasons for this late clinical referral are a lack of awareness among primary care providers, limited diagnostic and therapeutic facilities, as well as socioeconomic problems. Moreover, parents are reluctant to discuss sexual issues, even with medical professionals [28]. Thailand has started the multidisciplinary management of ambiguous genitalia in 1979 [27], while, in Semarang, Indonesia, a start was only made in 1999. 

Out of 88 patients, nine (10.2%) were related, spread over four families. There was no known consanguinity of the parents in any of the cases. Thus, in only nine patients, a familiar background suggestive of an inherited disease could be established. One family with 2 affected children and their cousin from mother's side had the same *AR* gene mutations (R840H). Identical mutations on *CYP21A2* were found in 2 siblings in one family (IVS2-12A>G).

Based on physical examination, chromosomal analysis, and hormonal data and in a limited set of patients imaging and laparoscopy, the patients were categorized in accordance with the current consensus statement [8]. A secondary root diagnosis was made in all patients; however, it should be noted that the secondary root includes male undermasculinization of unknown etiology, which was assigned in 20 patients (34%). However, we cannot rule out 5*α*-reductase deficiency in these patients. 

In 15 out of 22 patients with 46, XX DSD, the diagnosis CAH based on *CYP21A2* mutations was made, including two familial cases. As a result of the diagnostic procedure, 12 patients are presently under steroid treatment. Three patients remained untreated (parents' request): two of them are sibs, and one of them showed a rather severe form of virilization. This patient was raised as a male, and when the diagnosis of CAH was made at the age of 17 yrs, he chose to continue to live as a male after full explanation was given. The parents decided to leave the 46, XX CAH sib (age 3 years) also untreated and are raising this child as a male. Parents of the third patient did not choose for hormonal treatment for economic reasons. One patient with presumed CAH died one week after inclusion. Because of lack of diagnostic and treatment options, it is suspected that patients with 46, XX DSD may have died from a crisis before coming to medical attention due to salt loosing CAH [29, 30].

Interestingly, one patient at first thought to have CAH turned out to have an androgen producing ovarian tumor with the histology of Leydig cell tumor. This demonstrates the value of histological examination of abdominal lesions in these patients.

One patient was categorised as 46, XX, gonadal dysgenesis. Gene mutation analysis was done in a patient suspected of a glucocorticoid receptor defect, but no mutant sequence was found [31], and, in none of the four patients with the clinical diagnosis of Mayer-Rokitansky Syndrome, a *WNT4* mutation was detected [20]. 

Normal development and function of Sertoli cells and Leydig cells are essential for hormone-mediated sex differentiation of male internal and external genitalia.

In order to diagnose 46, XY DSD, determination of LH, FSH, gonadal steroids, AMH, and inhibin B levels is essential. Leydig cell activity is examined by hCG stimulation. In our patients, measurement of testosterone precursors such as androstenedione, 17-OH progesterone, progesterone, and DHEA did not give evidence of a testosterone synthesis disorder such as 17*β*-HSD or 17–20 lyase deficiency [5, 7]. Only in two patients, no rise of testosterone and its precursors was observed after hCG stimulation, suggesting an LH receptor defect. However, no mutation of the LH receptor gene was found [32]. In most of the patients with the clinical phenotype of Leydig cell hypoplasia, no causative mutations are found [6]. 

In prepubertal patients, low AMH levels indicate malfunctioning Sertoli cells in the testis. The best marker to evaluate the presence of functional testis after puberty is inhibin B [33]. Circulating concentrations of AMH remain high until puberty when they fall in response to the effect of testosterone. For this reason, we decided to categorize normal AMH values based on testosterone levels [34]. We confirmed that increased AMH levels after puberty (testosterone level >6 nmol/L) are suggestive for a disorder of androgen action or synthesis. The combination of high LH and testosterone levels in undermasculinized patients also supports a defect in androgen action. However, in only 12 (25%) of the in total 48 patients with 46, XY DSD with clinical and hormonal features compatible with altered androgen sensitivity, an AR mutation was found. Two of the mutations were pathogenic, and four mutations were unclassified variants which in later investigation were found to be pathogenic [23]. All of these patients showed the typical features of partial androgen insensitivity. It is noteworthy that 11 patients are being raised as males and only one as female. In none of the patients classified as severe (penoscrotal) hypospadias, an AR mutation was found. 

All patients with chromosomal DSD had a chromosomal mosaicism. Three patients had a Klinefelter variant; all of them were raised as males.

Although imaging procedures are highly informative for the establishment of a second root diagnosis, sonography was only performed in 28/88 patients (31.8%) and a diagnostic laparoscopy or cystoscopy was done in only nine of the patients (10.2%). Limited facilities and economical problems are the main reason for these relatively low numbers. In 30/58 patients with 46, XY DSD (51.7%), genital surgery was performed such as hypospadias correction, gonadectomy, and mastectomy. The rest of the patients (*n* = 28) remained untreated mostly for economic reasons. Some patients or parents refused the advice of the gender team and just dropped out (*n* = 9). One assumption is that the cultural reasons are of influence. Decision making is not just based on what is recommended by the doctor but is influenced by the family. 

The risk of developing a malignant germ cell tumor is increased in patients with DSD containing Y chromosomal material, known as the gonadoblastoma locus on the Y chromosome (GBY). This phenomenon is probably related to the expression of the *TSPY *gene on the Y chromosome [19]. It is important to mention that a nonscrotal position of the gonad increases this risk. Proactive clinical interference, like orchidopexy, biopsy, or even gonadectomy, is recommended in patients with 46, XY DSD with maldevelopment of the testes (with or without known gene mutation such as *WT1*) and, in addition, in patients with PAIS, especially when the gonads are in a nonpalpable position [10]. It is noteworthy that, in our small sample of four gonadectomized patients, already one patient with 46, XY DSD had developed carcinoma *in situ* (CIS). This is the known precursor of malignant germ cell tumors, which will progress to invasiveness in about 70% within seven years. A biopsy was not performed in all patients at risk because of the limited resources. This raises the question whether a prophylactic gonadectomy in all patients at risk for a malignancy should be performed. At the moment, research is conducted focussing on the identification of factors to estimate the actual cancer risk in the individual patient to prevent unnecessary prophylactic gonadectomy [10]. 

A point of debate is the inclusion of the histology and the genetic analysis in a diagnostic workup. Of course mutation analysis provides confirmation, whereas the histology is also an important prognostic parameter as a base for further treatment. We used the tertiary root only if a mutation was found or if gonadal histology was known. This was the case in 29 out of 88 patients (32%).

In conclusion, in daily practice the implementation of the current consensus statement in a resource-poor environment is very difficult. Especially the tertiary root classification that is based on molecular genetic or histopathology diagnostics is in many cases not feasible. 

Therefore, we recommend the following stepwise approach: as a first step, a careful clinical evaluation, karyotyping of peripheral blood and sonographic imaging of the internal genitalia should be performed in all patients. 

Subsequently, in the 46 XX patients, rapid determination of 17-hydroxyprogesterone in serum or saliva [35] is needed in the first week of life in order to recognize a salt-loosing CAH and prevent a life-threatening crisis. An increased level is highly suggestive of the diagnosis CAH and needs to be followed by immediate initiation of lifesaving treatment. In patients with 46 XY or Y chromosome containing DSD, determination of gonadotropins, testosterone, DHT, inhibin B, and AMH is to be performed. Dependent on age and stage of puberty, a second root working diagnosis can be made allowing gender assignment and planning for further diagnostic procedures and management in collaboration with global DSD centers of excellence. 

This implies the need for education of primary health care workers on how to recognize DSD as a clinical feature that requires urgent assessment to prevent morbidity and mortality in some cases. Protocols on referral pathways should be implemented. 

Unfortunately, in Indonesia, several factors such as patients' and general society's opinion on DSD problems, economic background of DSD patients, and lack of access to health insurance can affect the complex management of DSD in a negative way.

## Figures and Tables

**Table 1:** Phenotype (Quigley stage) in correlation with the DSD classification and sex of rearing. NA: not assigned, patient died after inclusion but before gender assignment.

Classification	Consanguinity %	Quigley stage							Sex of rearing	
		1	2	3	4	5	6	Male	Female	NA
46, XX DSD	0		3	5	6	4	4	2	19	1
46 XY DSD	0	4	22	22	6	1	3	45	13	
Chrom. DSD	0	1	3	0	2	0	2	5	3	

**Table 2:** Karyotype of patients with chromosomal DSD.

Karyotype	Number
XXY/XY	2
XY/X	2
XX/XXq-	2
XY/XX	1
XX/XXY	1

Total	8

**Table 3:** Additional clinical data in patients with known histology results.

Number	Age	Gender	Karyotype	Quigley stage	Hormonal analysis	Imaging	Surgery	Mutation	Histology	Diagnosis
1	33	Female	46 XY	4	Very high testosterone with slightly increased values of gonadotropins	No abnormalities	Laparoscopy	No	Ovarian Leydig cell tumor lacking histological signs of malignancy	Disorder of androgen excess

2	23	Female	46 XY	6	High basal testosterone level	No abnormalities	Gonadectomy	AR neg	Leydic cell hyperplasia and atrophy of most seminiferous tubules but no evidence of CIS	Disorder of androgen action

3	13	Male	46 XY	3	High basal testosterone level	Gonads not in situ* no uterus and adnexa 1/3 vagina present	Laparoscopy gonadectomy	AR neg	CIS	PAIS

4	3	Male	46 XX/XY	2	hCG test: good response testosterone	No abnormalities	Gonadectomy	SRY neg	Ovarian tissue with multiple cysts including primordial follicles and granulosa cells	Gonadal dysgenesis

*Gonads already removed before ultrasound.

**Table 4:** Final classification.

DSD classification	Number of patients	Total
*46, XX DSD*		

Disorder of androgen excess, other	2
Disorder of androgen excess, 21 hydroxylase deficiency	15
Defect of mullerian development, other	4
Disorder of gonadal development, ovotesticular DSD	1	22

*46, XY DSD*		

Disorder of gonadal development, other	9
Disorder of androgen action, PAIS	12
Disorder of androgen action, other	17
Male undermasculinisation of unknown aetiology	20	58

*Chromosomal DSD*		

46 XX/46 XY DSD, disorder of gonadal development, ovotesticular DSD	1	
47 XXY DSD, disorder of gonadal development, other	3
45X/46XY DSD, disorder of gonadal development, other	2
Other, disorder of gonadal development, other	2	8
